# Vocal Hinderances

**Published:** 1899-04

**Authors:** Dwight L. Hubbard

**Affiliations:** New York


### International Dental Journal.

Vol. XX. April, 1899. No. 4.

### Original Communications.[1]

### VOCAL HINDERANCES.[2]

BY DWIGHT L. HUBBARD, M.D., NEW YORK.

Demosthenes stammered. He attempted to remedy the difficulty by going to the sea-shore to practise oratory aided by pebbles. His indefatigable persistence might well be imitated by those who aspire to the art either of singing or speaking. Much may be accomplished when circumstances are unfavorable. Demosthenes conquered and became great. His individuality, illustrated by untiring zeal and determination, was that which accomplished for him not only the foundation but the superstructure of proper voice-production. I say it accomplished *for him* a proper voice-production. A rule suitable for one may not be a rule suitable for all. Let me say here that I do not believe in particular methods. There is no method for all alike. There is, in a general way, *a* method; but it must in every case be modified by the anatomical and physiological construction of the vocal apparatus of each individual. The basic principles must be followed, but let it be borne in mind that as no two violins are exactly alike, so no two human vocal instruments are of the same volume or timbre. That which will

[1] The editor and publishers are not responsible for the views of authors of papers published in this department, nor for any claim to novelty, or otherwise, that may be made by them. No papers will be received for this department that have appeared in any other journal published in the country.

[2] Read before The New York Institute of Stomatology, January 3, 1899.

follow, therefore, will be subject to the modification stated. The aspiring violinist must learn how to hold the instrument and draw the bow, the rules for which are alike for all learners. But when ease in the mechanic art is accomplished, the individuality and quality of the instrument upon which the learner depends for the interpretation of his own soul must be acquired. Then he will discover that his interpreter has also a soul all its own. In like manner the human voice is subject to the same general laws, applicable to phonetics and acoustics. When interpretation is required, the laws governing its mechanism must of necessity be followed. This accomplished, the mind and soul speak through and by the means of the instrument, with necessary variation from set rules. The child-like wildness of a Calvé was not acquired; it was there in the beginning. The rules were inborn. The plaintive sweetness of a Melba was not an acquirement; it was there long before the voice spoke in the appeal of Marguerite to her God. The long inherited combination which lends the abandon or the self-consciousness, which is sweetness only when ignored, makes the rules and standards for the guidance of imitators. Rules necessitated by mechanics are conventional and for those who lack genius.

But to return. Certainly determination *is* essential. Many there are who *can* sing and will not. Greater is the number who cannot sing, but still they persist in the attempt to reproduce sections of the Wagnerian compositions or to experiment in Demosthenian oratory. The productions of great composers and writers must be interpreted intelligibly by the use of physio-mechanical apparati, and sometimes, if I may use the phrase, dynamic force, but still with the breathings of a whispered confidence. Grand, subtle, and infinitely divine ideas may be conveyed provided the genius is there and, not less important, all the mechanical necessities are fulfilled. The true method, therefore, is that one which combines all these qualities.

As of the greatest importance, I would speak first of the relationship existing between the general health and these organs of special sense. The artificial voice of the educated mute bears testimony to the necessity for good hearing. The blind speak and sing with coldness. Blue people with torpid livers make themselves disagreeable with melancholy strains and words. Those of uric acid diathesis are nervous and spasmodic in their renderings. Splenic people speak with a nonchalant air and vent their spleen upon the unoffending. Dyspeptics are morose, express even their own ideas incorrectly, and are incapable of speaking for others. People subject to inflammatory conditions of any part of the body, creating a central nervous disturbance, are unfit for society and lose their personal magnetism. Those suffering pain or mental depression communicate the same with all that is said or done. So we might go through the category of general pathology and find that true happiness and the ability to lend ourselves to the highest and noblest in life, especially to those who *truly* live, consists chiefly in health.

Cursorily, we shall consider a few general conditions which influence the vocal mechanism. First in importance is the alimentary tract. Here the glandular system must be taken into consideration. Good sewerage is a necessity to the proper function of all glands. Although this principle applies to such glands as the liver, the spleen, the pancreas, the kidneys, the peptic glands, and others of like character, and also to the whole lymphatic system, we must not forget that the mucous membranes of the body, especially those of the respiratory tract, are abundantly supplied with such structures. The basement and ciliated epithelium of the nasal mucous membrane, the lymphoid tissues standing guard almost completely around the nasopharynx and pharynx, the desquamating scales of the oral cavity, the lubricating mechanism of the laryngopharynx and trachea to its bifurcation, would be perfectly useless in their offices without the monitors which stand guard between them and the peripheral nervous system,—viz., the microscopic glands. Here we reach the secret of the relationship between all general conditions of the body and special functions. Action and reaction explain many of the problems in obscure disease.

Diseases of the teeth alone have no small significance in their relation to general health. The studies of scientific dental men have proceeded so far beyond the mere consideration of the teeth that there is much discussion as to whether it would not be better to change the word "dentistry" to "stomatology," and for those who are prepared by dental college teaching of to-day to change the word "dentist" to "stomatologist."

Oral pathology merits our careful consideration. The sooner we recognize the value of the researches of such men as Black, Williams, Evans, and others of the dental profession, so much the sooner shall we receive light upon a subject left too much in the dark, but upon which we may well turn the electricity of modern thought and research. Medical men leave much of this to the dentist, and take it for granted that the rhinologist will take care of the parts overlooked. This ought to be, but I am sorry to say that the mouth does not receive the attention it deserves from even the rhinologist, who ought also to be a stomatologist. Dentists up to the present time have been totally unable to appreciate the general relationship of the pathology of the oral cavity to the general system, or the comprehensive application of physiology to the first and most important act of the organs of digestion and the final assimilation of its products. The bridge is being broken down, and the education of the modern dentist is running the medical man a close race in the more minute pathology of oral diseases and the importance of their effects, from the pharyngeal constrictors to the final assimilation of nutriment and the expulsion from the system of useless material. It will be conceded by all that a normal condition of the mouth and of the mechanism of mastication are essential to good digestion.

What are abnormal conditions? They may be cited under the head of diseased states of the upper respiratory tract, improperly, of course, including the mouth; catarrhal conditions of the head cavities, such as the frontal, sphenoidal, and maxillary sinuses and the ethmoidal cells. These are productive of gastric and intestinal disturbances. A catarrhal secretion from these will interfere with the normal preparation of the food by the stomach as surely as water flows down hill. The old-time and homely comparison just used is not used without significance. Catarrhal secretions always *flow down hill.* The nasal plane inclined backward from the anterior nares to the choanæ is the channel for frontal, sphenoidal, and ethmoidal secretions. The nasopharynx receives them and, especially during sleep, carries them on to the constrictors and into the stomach. It is not necessary to dwell upon such semi-purulent and acid secretions as are evidenced by means of the microscope and litmus-paper. Purulent secretions having an acid reaction are not normal. Rebellion is the result. The very initial stage of digestion is interfered with, and the more remote results are manifest. Couple with the catarrhal conditions above described the carious and pathogenic germ-carrying deposits upon the teeth, and we have all the elements which may be productive of malassimilation and malnutrition. As the result not only of carious conditions of the teeth but of disease of the whole oral cavity, different forms of micro-organisms are ingested. The various forms of yeast fungi, the leptothrix buccalis, the oidium albicans, and the spirillum of Müller are found in this locality. The last is found *particularly* in carious teeth.

According to general rule, the process of cell-formation is a very rapid one. Bacilli, whether rigid or flexible, motile or non-motile, have a very rapid development. According to Cohn, to complete the process of segmentation and for a new cell to attain the size of a parent cell would require only one day to produce sixteen million cocci; at the end of two days, two hundred and eighty-one million would be produced; while at the end of the third day the enormous number of forty-six trillions will have been reached. But such numbers are entirely beyond our comprehension. Suffice it to say that infection is dangerous from whatever locality it may come and from whatever cause it may arise. We have among the micro-organisms found in the buccal cavity the oidium lactis (milk mould), the leptothrix buccalis and gigantiæ, and various forms of organisms found also in other parts of the body. It is not necessary to enumerate them. The effect upon digestion is the thing to be considered. The same acid medium which invited their presence is that which, in the upper alimentary tract, keeps them alive and active. They are not destroyed by the digestive juices, but are nourished by this medium, in which they rapidly multiply and thrive. Stomach and duodenal digestive preparation, thus interfered with, is fruitful in the untoward results evidenced in the important digestive functions below. Assimilation cannot be perfect under these conditions. What are the results? First, rebellion manifested by constipation, with resultant flatulence. This is nearly always traceable to improper preparation of the food by the sentinels of good digestion,—viz., the teeth and buccal secretions. What is the remedy, —intestinal stimulants and antacids? Yes, when correction of primary causes is impossible. But the class of accepted remedies just named is an admission of primary causes. Second, too great an expenditure of vital nerve-force from the aforesaid rebellion results in irritation of the intestinal mucous coats, and diarrhœa is the result. Such, in a general way, is the history of disturbed digestion. It is not a "tempest in a teapot," but a reasonable relationship between cause and effect. Fundamental principles are the factors which make the modern practice of medicine scientific.

Leaving generalizations, it is proper to consider the oral cavity and adjacent structures in their relation to the larynx, at the same time exercising that optimism, without which no one can be prepared to practise.

What is voice-production? Garcia cleared up this much-discussed question by his discovery of the laryngoscope. That I may not be too presuming, I quote from Bosworth: "He [Garcia] demonstrated conclusively that the action of the larynx is really that of a reed instrument, and that the column of expired air is thrown into sonorous vibrations by the vocal cords. For the accomplishment of this purpose the cords are brought into apposition in the median line, and, being held firmly in position, are rendered tense by muscular action when the air is forced through by the respiratory muscles of the chest in such a way as to throw their edges into vibration. In this manner the column of air in the upper tract is also thrown into vibration and, as has been stated, converted into articulate language by the movements of the tongue, lips, palate, etc."

I have thus fully quoted from an eminent authority to recall to you the pure mechanics of voice-production and to show that co-ordination between the oral, nasopharyngeal, and nasal structures and the interior of the larynx is an absolute necessity. In any diseased state involving the mouth, throat, nose, or sinuses, co-ordination is interfered with, whether the interference be caused by any of the different forms of stomatitis or by any mechanical or pathological condition of the oropharynx, nasopharynx, nose, etc. A healthy condition of the soft palate is of the greatest importance. The mucous membrane of the larynx follows the rule which governs mucous membranes lining other portions of the air-tract, in that it is covered with columnar epithelium, with the exception of the vocal cords, which are covered with squamous epithelium. Although ciliated epithelium is nature's provision against the attacks of invaders, it becomes powerless when *inflammation* attacks the mucous membrane upon which it is fixed. In the state of fixation thereby produced it furnishes a ready ground for inflammation and the retention of almost any form of micro-organism. In fact, the inspiratory act takes such invaders to their choice lurking-places. Some pathogenic micro-organisms which are common to the mouth are the bacterium lactis, the oidium lactis, and the leptothrix buccalis, all of which have to do directly with caries. The first two named are naturally more frequently found in the mouths of children. Many cases of so-called pseudomembranous croup are undoubtedly directly or indirectly caused by the presence of these organisms. We might say that under these circumstances they become really "saprophytic" germs. In what I have just said it is my desire merely to call your attention to the possibility, or even the probability, that such conditions make a favorable soil in the respiratory tract for either direct extension of inflammation, or as indirect causative agents by means of chemical changes.

I wish to say a word about latent germs.

The foes from within are as numerous as the foes from without. That man is his worst enemy is well illustrated in the pathological principle that the acquired and entertained micro-organisms which have found a lodgement within the body lie dormant only so long as a favorable chance does not present itself for their further entertainment, development, and growth. This is the explanation of the following phenomenon, which we often find: Wounds, although externally made perfectly aseptic, develop pus on account of pyogenic germs in circulation. In diseased conditions of the mouth another element of danger thus presents itself.

Why are syphilitic ulcerations of the vocal bands so common? Because this locality is the most exposed, and is the most direct point of attack by the inspired air which passes over existing secondary mucous patches in the oral cavity. Such ulcerations, if they occur at all, generally follow the mucous patches. Laryngeal syphilitic ulceration is the bridge between the secondary and the tertiary stage.

I would not leave the subject without referring to hoarseness and loss of voice through "traumatism." I do not use the word in its ordinary sense, but with a view to the non-pathologic character of many cases. Nasal and nasopharyngeal stenosis cause mouth-breathing, and from lack of *moistened* air produce a traumatic inflammation,—*e.g.*, in atrophic rhinitis. Atrophy of the glands of the mucous membranes of the nasal cavity invariably produces unnatural changes in the voice. Catarrhal secretions from hyperplastic, hypertrophied, or atrophied tissue in the upper respiratory tract cause, from the nature of the principles of mechanics, either a muffled, throaty, hoarse voice, or, through muscular inability from long-continued over-taxation, loss of voice.

In a paper read recently before the Northeastern Dental Association on the subject of "Voice and Artificial Dentures," I tried to show that the voice would be modified by plates, etc. How much more will abnormal hypertrophies, which cannot receive prosthetic skill, cause trouble in tone, volume, quality, and timbre. The velum palati is, as already mentioned, one of the most important organs of speech. Its free muscular play must not be interfered with either by inflammatory changes, of whatever nature, or by the presence of mechanical obstruction.

To summarize:

As stomatologists, we must not forget the *whole* body, nor confine ourselves in diagnosis or treatment to the oral cavity, forgetting the intimate relationship between it and the respiratory tract proper.

